# Safety and efficacy of corticosteroids in ARDS patients: a systematic review and meta-analysis of RCT data

**DOI:** 10.1186/s12931-022-02186-4

**Published:** 2022-11-04

**Authors:** Xinyan Chang, Shaojun Li, Yueqiang Fu, Hongxing Dang, Chengjun Liu

**Affiliations:** 1 Department of Intensive Care Unit, Children’s Hospital of Chongqing Medical University, National Clinical Research Center for Child Health and Disorders, Ministry of Education Key Laboratory of Child Development and Disorders, 136#, Zhongshan Er Road, Yuzhong, Chongqing, 400014 China; 2 Department of Emergency, Children’s Hospital of Chongqing Medical University, Chongqing, China; 3 Chongqing Key Laboratory of Pediatrics, Chongqing, China

**Keywords:** Acute respiratory distress syndrome, Corticosteroids, Children, Adults, Meta-analysis

## Abstract

**Purpose:**

Acute respiratory distress syndrome (ARDS) is an acute and critical disease among children and adults, and previous studies have shown that the administration of corticosteroids remains controversial. Therefore, a meta-analysis of randomized controlled trials (RCTs) was performed to evaluate the safety and efficacy of corticosteroids**.**

**Methods:**

The RCTs investigating the safety and efficacy of corticosteroids in ARDS were searched from electronic databases (Embase, Medline, and the Cochrane Central Register of Controlled Trials). The primary outcome was 28-day mortality. Heterogeneity was assessed using the Chi square test and *I*^2^ with the inspection level of 0.1 and 50%, respectively.

**Results:**

Fourteen RCTs (n = 1607) were included for analysis. Corticosteroids were found to reduce the risk of death in patients with ARDS (relative risk (RR) = 0.78, 95% confidence interval (CI): 0.70–0.87; *P* < 0.01). Moreover, no significant adverse events were observed, compared to placebo or standard support therapy. Further subgroup analysis showed that variables, such as adults (RR = 0.78; 95% CI: 0.70–0.88; *P* < 0.01), non-COVID-19 (RR = 0.71; 95% CI: 0.62–0.83; *P* < 0.01), methylprednisolone (RR = 0.70; 95% CI: 0.56–0.88; *P* < 0.01), and hydrocortisone (RR = 0.79; 95% CI: 0.63–0.98; *P* = 0.03) were associated with 28-day mortality among patients who used corticosteroids. However, no association was found, regarding children (RR = 0.21; 95% CI: 0.01–4.10; *P* = 0.30).

**Conclusion:**

The use of corticosteroids is an effective approach to reduce the risk of death in ARDS patients. However, this effect is associated with age, non-COVID-19 diseases, and methylprednisolone and hydrocortisone use. Therefore, evidence suggests patients with age ≥ 18 years and non-COVID-19 should be encouraged during the corticosteroid treatment. However, due to substantial differences in the use of corticosteroids among these studies, questions still remain regarding the dosage, optimal corticosteroid agent, and treatment duration in patients with ARDS.

**Supplementary Information:**

The online version contains supplementary material available at 10.1186/s12931-022-02186-4.

## Introduction

Acute respiratory distress syndrome (ARDS) is a serious condition, which is characterized as refractory hypoxemia caused by various factors and is usually secondary to a variety of lung diseases or extrapulmonary conditions, such as pneumonia, drowning and non-pulmonary sepsis [1]. It could lead to respiratory failure, critical illness, and even death, particularly in children [2,3]. A previous study showed that ARDS is estimated to be prevalent in 10.4% of patients requiring intensive care unit (ICU) care [4]. Although mechanical ventilation and fluid management have proved their efficacy for ARDS treatment in recent history, effective treatment remains a challenge, such as tidal volume, high positive end-expiratory pressure, advanced infection, and fewer drugs [5–7].

The concept of ARDS was first proposed in 1967 by Ashbaugh et al. [8]. ARDS is known as an excessive inflammatory response which is caused by damage to the alveolar capillary endothelial cells and alveolar epithelial cells, resulting in pulmonary interstitial edema [4], alveolar edema, airway occlusion, and alveolar atrophy [1,9–11]. Corticosteroids were considered as one of the potential therapeutic drugs[8] as early use of corticosteroids were shown to reduce systemic inflammatory response and accelerate the recovery of pulmonary infection [4]. This may be related to the role of glucocorticoids, which can inhibit the synthesis of cytokines and reduce the proliferation and regulation of T cells, macrophages and others [12].

Currently, as few as 30–35% of children diagnosed with ARDS are administered steroids in clinical practice [13]. However, in adults, many randomized controlled trials (RCTs) using corticosteroids have been performed [14,15]. Unfortunately, there is still a lack of conclusive evidence and guidelines for the use of corticosteroids in ARDS patients [16]. This may be due to the conflicting data between recent and previous studies [17–22], and a significant heterogeneity being found among the results of the RCTs [18,23–27]. In addition, the efficacy of corticosteroids has been found to be associated with several clinical variables, and in some cases, corticosteroids are not recommended routinely [1,28].

Therefore, the safety and efficacy of corticosteroids are still unclear. Hence, in this study, we performed a systematic review and meta-analysis to assess the safety and efficacy of corticosteroid administration in patients with ARDS, compared to those without corticosteroids. In addition, subgroup analysis of glucocorticoid regarding age, etiology, type of corticosteroids, and treatment duration was also performed.

## Material and methods

### Databases and search strategy

The study was registered on PROSPERO (CRD42022314505) and performed according to the PRISMA statement [29]. We systematically searched Embase, Medline, and the Cochrane Central Register of Controlled Trials (from 1963 to March 15th, 2022). The terms, including *corticosteroids, hydrocortisone, methylprednisolone, dexamethasone, acute respiratory distress syndrome, ARDS, and randomized controlled trial*, were searched alone or in combination without language restriction. Only human studies were included and the search strategy is detailed in the Supplementary Materials.

### Eligibility criteria

Eligible RCT studies were searched and selected for analysis according to the criteria for participants, interventions, comparators, outcomes, and study design. Patients were eligible if they were diagnosed with ARDS (based on manifestations [8], the American-European Consensus Conference and the Berlin Definitions [3,7]) and aged > 28 days (onset). The intervention included any corticosteroid treatment and the comparator included standard supportive care (such as mechanical ventilation, antibiotics, and fluid replacement) and placebo administration [1].

Two independent investigators (X.C. and S.L.) screened the titles and abstracts of the included articles. Any discrepancies between the two investigators were resolved through discussion, or decided by the third investigator (C.L.). When the data of the included articles was missing, the corresponding authors were contacted to obtain the data. The risk of bias and methodological quality were assessed using the Cochrane Risk of Bias Tool 2.0 [30].

### Data collection process

The primary outcome was 28-day mortality. In-hospital or ICU mortality was used to calculate the pooled 28-day mortality, unless actual 28-day mortality rates were reported or obtained from primary authors [31]. Secondary outcomes included ICU mortality, in-hospital mortality, 60-day mortality, length of ICU stay, length of in-hospital stay, and ventilation-free days to day 28. Adverse events included hyperglycemia and gastroduodenal bleeding. Patients with chronic diseases requiring long-term corticosteroid and immunosuppressive therapy were excluded. Data was collected by the two investigators (X.C. and S.L.) separately using a standardized table and any discrepancy was resolved through discussion, or decided by the third investigator (C.L.).

### Subgroup analysis

Subgroup analysis was performed on the following variables: children/adults (children, < 18 years, and > 28 days; adults, ≥ 18 years); etiology (Coronavirus disease 2019 (COVID-19) or non-COVID-19); corticosteroid type (hydrocortisone, dexamethasone, methylprednisolone, etc.); treatment duration (≤ 7 days, 8–14 days, and ≥ 15 days); and methylprednisolone dose (high, > 2 mg/kg/d; low, ≤ 2 mg/kg/d).

### Data synthesis

The statistical analysis was performed using Review Manager, version 5.4.1 (Cochrane Collaboration), and Stata, version 16.0 (College Station, Texas, USA). Heterogeneity was assessed using the Chi square (*Chi*^2^) test and the *I*^2^ test with an inspection level of 0.1 and 50%, respectively. If the *I*^2^ was < 50% and *Chi*^2^ > 0.1, fixed-effects models were employed for the analysis. Otherwise, random-effects models were used. When there was a difference between the two, the *Chi*^2^ test results follow the *I*^2^. Dichotomous data was analyzed using the Mantel–Haenszel method, and the pooled risk ratios (RR) and corresponding 95% confidence intervals (CI) were then calculated. Continuous data was analyzed using the Inverse Variance method and expressed as mean difference (MD) and 95% CIs. If only median and interquartile range were available, mean and standard deviation were estimated using the Mean–Variance Estimation [32]. Potential publication bias was assessed by funnel plot, Egger's test, and Begg's test [33].

## Results

### Study selection and study characteristics

**Fig. 1:**
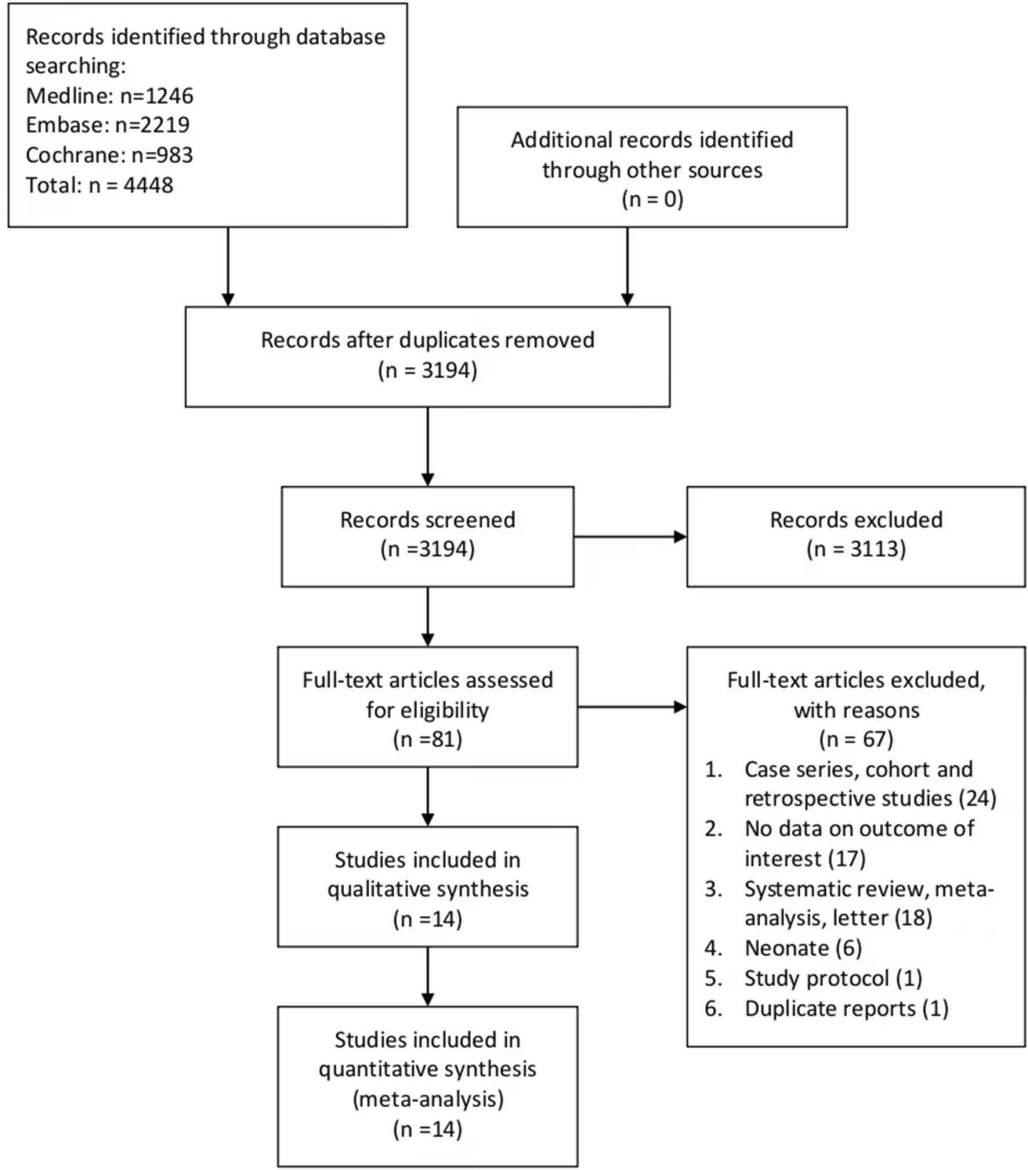
Selection process for RCTs included for the analysis

A total of 4448 potentially eligible records were found through a comprehensive literature search (Additional file 1: Table S1). The duplication was performed using MedRef (Jinyetiancheng, Beijing, China) [34]. After excluding duplicates and reviewing the titles and abstracts, 81 studies remained in the study. After reading the full text, 14 studies [14,15,17,18,23–26,35–40] met the eligibility criteria, were included for the final analysis and had a sample size ranging from 24 to 393 (Fig. 1).

The 14 studies were published between 1963 and 2022, including 1607 participants. Of these, nine studies were multicenter [14,17,18,23,25,26,37–39], ten studies used placebo as controls and another four studies used standard therapy [15,17,18,35] Seven studies [14,23,25,26,35,39,40] used methylprednisolone, four [24,36–38] used hydrocortisone, and three [15,17,18] used dexamethasone. Treatment duration ranged from 1 to 32 days. The corticosteroid dose varied widely among the included studies, ranging from 0.125 to 120 mg/kg/d of methylprednisolone (or equivalent; Additional file 2: Table S2).

### Risk of bias and quality of evidence

First, seven studies were considered as having a low risk of bias [14,18,24–26,38,39]. Second, another seven studies were assessed as having low risk, regarding “deviations from intended interventions”, “missing outcome data”, “measurement of the outcome”, and “selection of the reported results category”. Among them, allocation concealments were not clearly described in six studies, which were assessed as unclear risk [15,23,35–37,40]. In addition, there was an open RCT without allocation concealment, which was considered to have a high risk of bias [17] (Additional file 1: Fig. S1).

### Primary outcomes: 28-day mortality

**Fig. 2:**
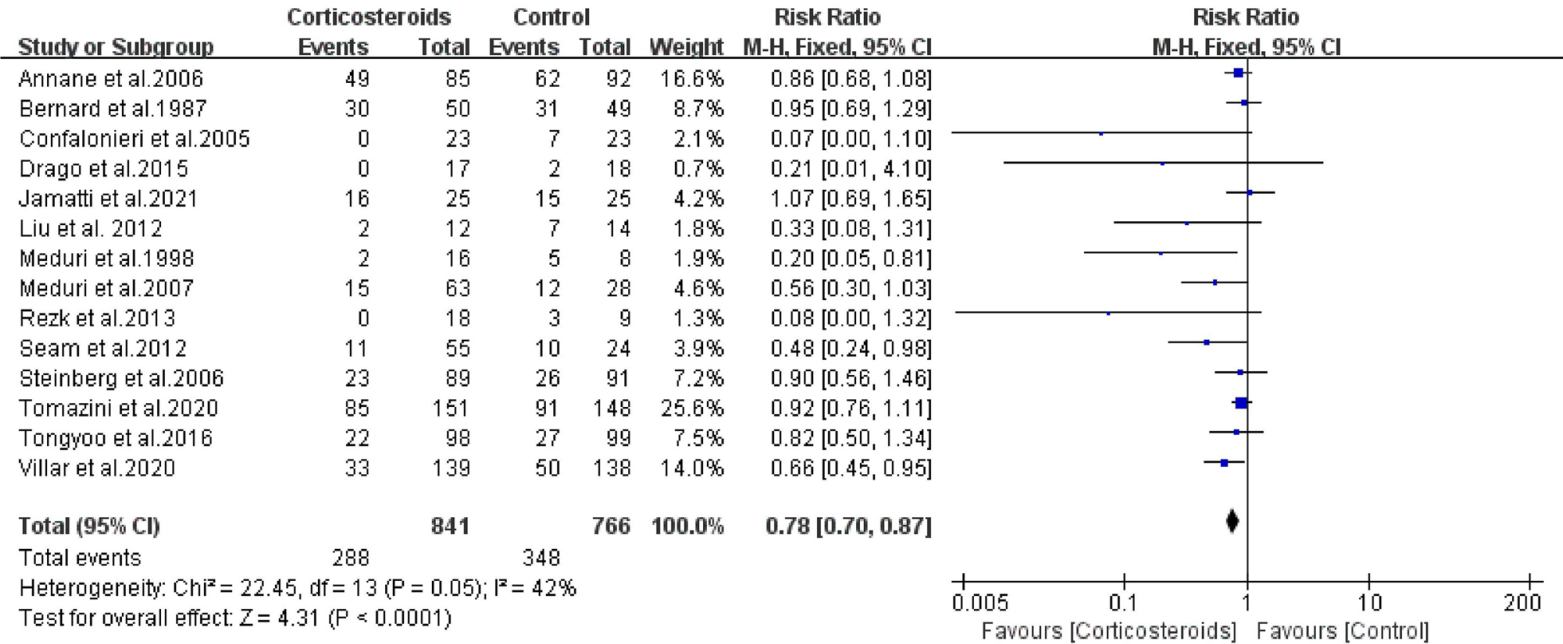
The effect of corticosteroids on Mortality at 28 days among patients with ARDS

Twenty-eight-day mortality was reported in fourteen studies (n = 1607). Overall, 28-day mortality was reported in 34.2% of those taking corticosteroids and 45.4% of those without corticosteroid use. Hence, the RR is estimated at 0.78 (95% CI: 0.70–0.87; *P* < 0.01), which revealed an association between corticosteroid therapy and 28-day mortality (Fig. 2). Therefore, it was demonstrated that corticosteroid use could decrease the risk of 28-day mortality. No heterogeneity between the studies was found according to *Chi*^2^ following to *I*^2^ (22.45 and 42%, respectively).

### Secondary outcomes

Four studies [14,18,37,40] reported ICU mortality, five studies [18,23,25,37,38] reported in-hospital mortality, and four studies [14,18,24,38] reported 60-day mortality. Corticosteroids were associated with in-hospital mortality (RR = 0.68, 95% CI: 0.47–0.98; *P* = 0.04) and ICU mortality (RR = 0.67, 95% CI: 0.45–0.98; *P* = 0.04), but not 60-day mortality (RR = 0.73; 95% CI: 0.50–1.06; *P* = 0.1). Therefore, we found that corticosteroid use could decrease the risk of in-hospital and ICU mortality. However, heterogeneity existed between the studies reporting on in-hospital mortality (*Chi*^2^ = 8.33, *I*^2^ = 52%), ICU mortality (*Chi*^2^ = 6.96, *I*^2^ = 57%), and 60-day mortality (*Chi*^2^ = 6.3, *I*^2^ = 52%) (Additional file 1: Fig. S2).

Six studies ^[[[[17,18,23–25,36]]]]^ (n = 925) provided data on ventilation-free days at 28 days. Pooled analysis suggested a significant increase of ventilation-free days at day 28 (MD = 3.53 days; 95% CI: 2.32–4.74; *P* < 0.01), and no heterogeneity among studies (*Chi*^2^ = 7.26, *I*^2^ = 31%) was found (Additional file 1: Fig. S3). This means that the corticosteroid used could reduce the dependence of ventilation. There was no association between corticosteroid use and duration of hospital stay (MD = − 4.19 days; 95% CI: − 13.49–5.12; *P* = 0.38) and ICU stay (MD = -2.90 days; 95% CI: − 10.42–4.62; *P* = 0.45). However, high heterogeneity among studies was found in in-hospital stay (*Chi*^2^ = 84.26, *I*^2^ = 96%) and ICU stay (*Chi*^2^ = 219.62, *I*^2^ = 99%) (Additional file 1: Fig. S4).

### Adverse events

The incidence of hyperglycemia (RR = 1.11; 95% CI: 1.0–1.23, *P* = 0.05) and gastroduodenal bleeding (RR = 1.34; 95% CI: 0.51–3.51; *P* = 0.56) was not statistically different between the groups. There was no heterogeneity found among the studies in hyperglycemia (*Chi*^2^ = 2.41, *I*^2^ = 0%) and gastroduodenal bleeding (*Chi*^2^ = 1.35, *I*^2^ = 0%) (Additional file 1: Fig. S5).

### Sensitivity analysis and publication bias

#### Subgroup analysis

**Fig. 3:**
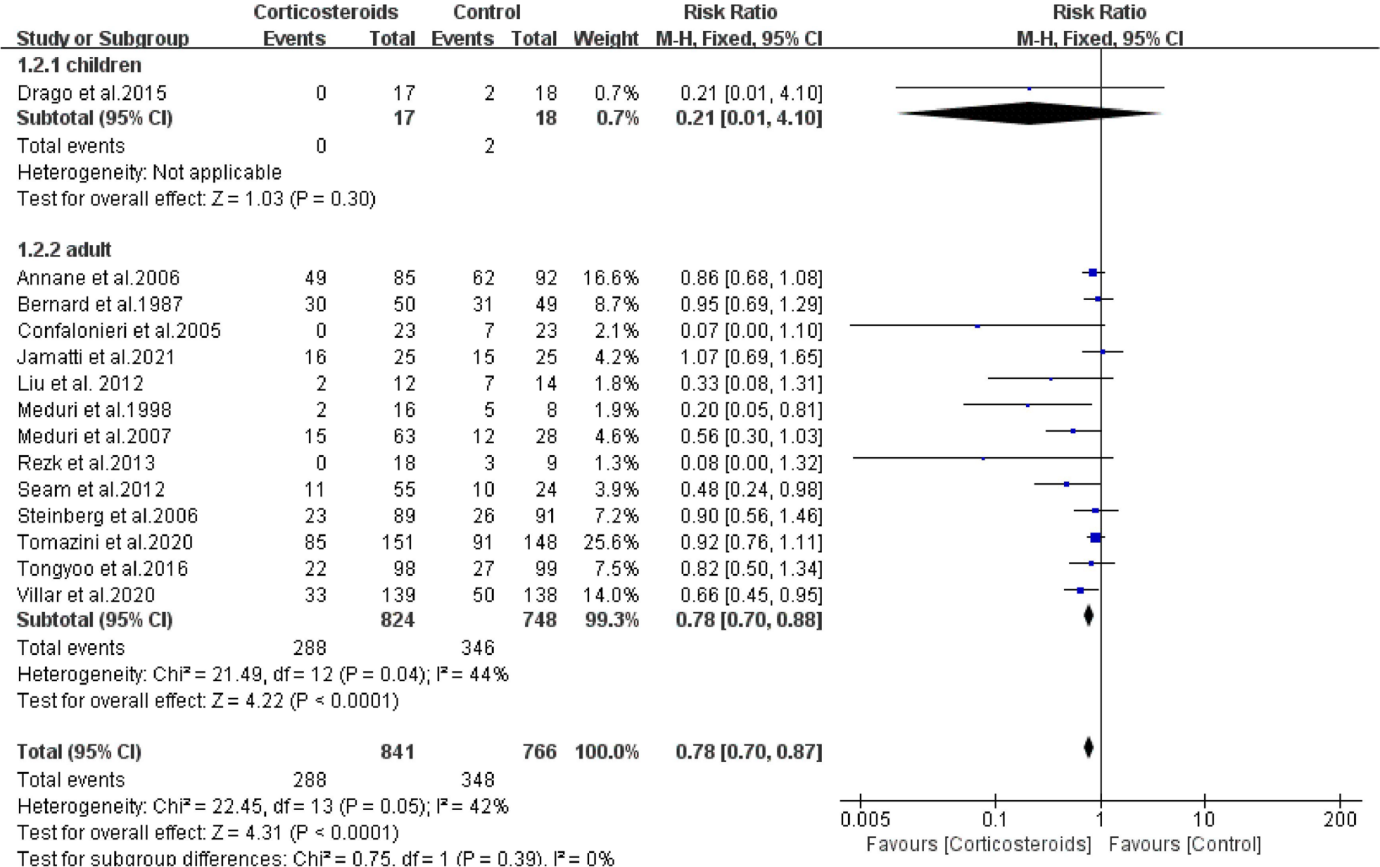
The effect of corticosteroids on Mortality at 28 days. Studies subdivided by adults and children

Adults/children: Ten studies included adult patients (n = 841, glucocorticoid group; n = 766, control group) and one included children (n = 17, glucocorticoid group; n = 18, control group). A fixed-effect model was used and the pooled data from the meta-analysis showed a significant association between mortality and glucocorticoid use in the adult subgroup (RR = 0.78; 95% CI: 0.70–0.88; *P* < 0.01), and there was no heterogeneity among included studies (*Chi*^2^ = 21.49, *I*^2^ = 44%). However, no significant association was found in the children subgroup (RR = 0.21; 95% CI: 0.01–4.10; *P* = 0.30) (Fig. 3).

**Fig. 4:**
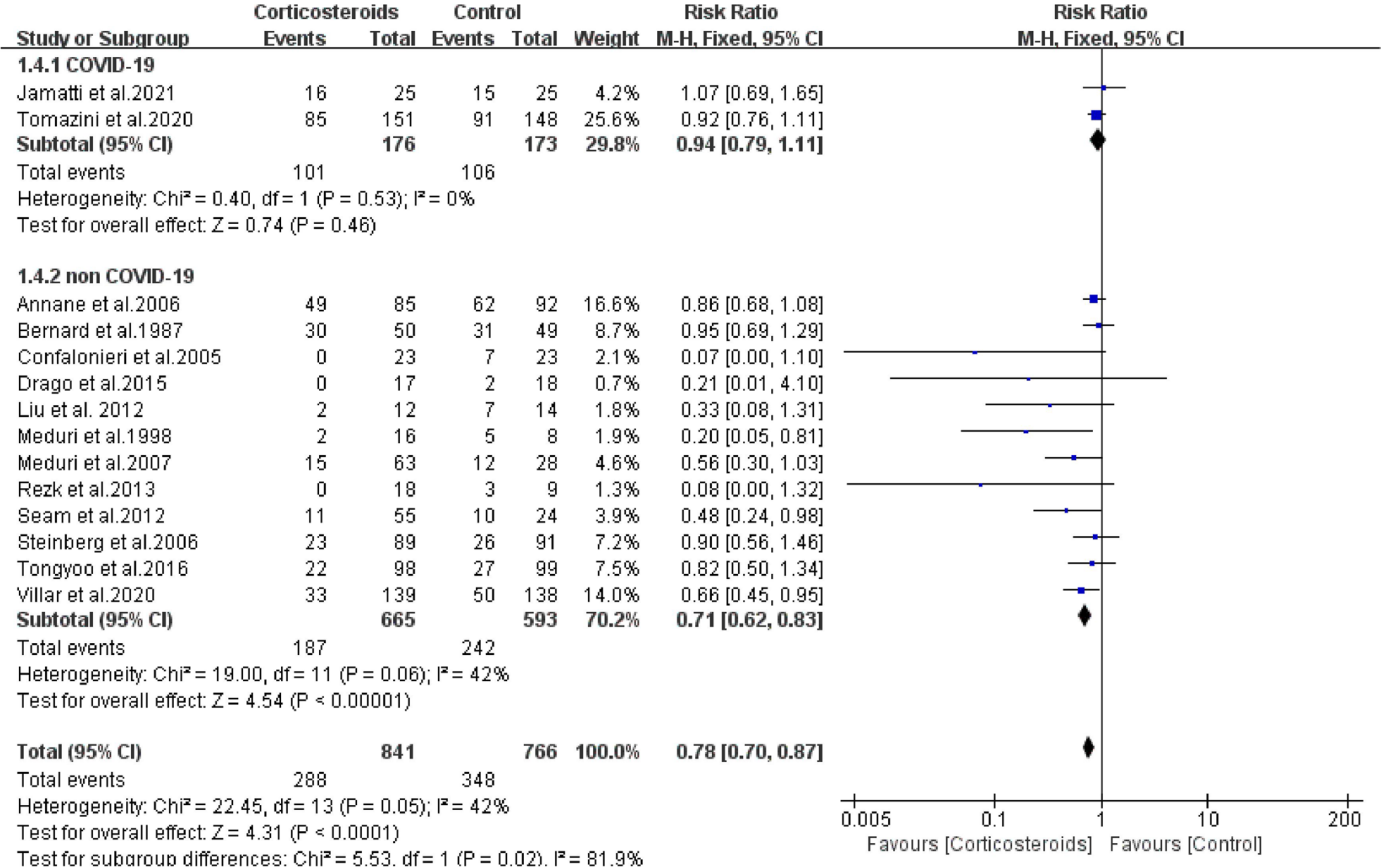
The effect of corticosteroids on Mortality at 28 days. Studies subdivided by COVID-19 status

Etiology: Two studies (n = 176, glucocorticoid group; n = 173, control group) included ARDS patients with COVID-19, and 12 studies (n = 665, glucocorticoid group; n = 593, control group) included ARDS patients without COVID-19. No beneficial effect of glucocorticoid treatment was found in the COVID-19 subgroup (RR = 0.94; 95% CI: 0.79–1.11; *P* = 0.46). Whereas, in the non-COVID-19 subgroup, corticosteroids were found to significantly reduce patient’s risk of mortality (RR = 0.71; 95% CI: 0.62–0.83; *P* < 0.01). Heterogeneity was not found among the studies, including in the COVID-19 subgroup (*Chi*^2^ = 0.4, *I*^2^ = 0%) and the non-COVID-19 subgroup (*Chi*^2^ = 19.0, *I*^2^ = 42%) (Fig. 4).

**Fig. 5:**
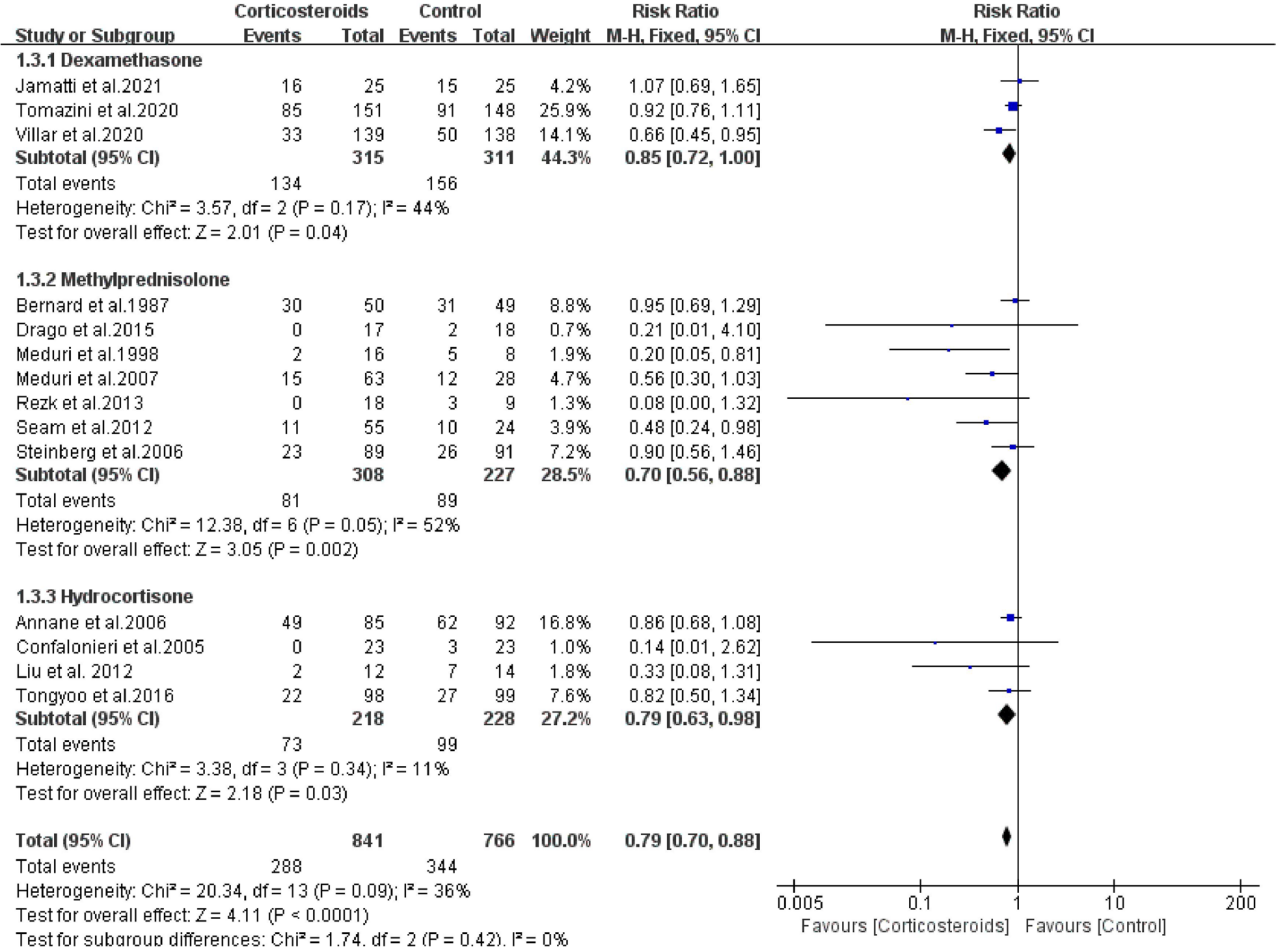
The effect of corticosteroids on Mortality at 28 days. Studies subdivided by corticosteroids types

Corticosteroid type: Methylprednisolone (RR = 0.70; 95% CI: 0.56–0.88; *P* = *P* < 0.01) was used in seven RCTs (n = 535) and hydrocortisone (RR = 0.79; 95% CI: 0.63–0.98; *P* = 0.03) in four RCTs (n = 446), both of which were found to significantly improve outcomes. However, three RCTs (n = 626) of dexamethasone (RR = 0.85; 95% CI: 0.72–1.00; *P* = 0.04) demonstrated a negative result. There was no heterogeneity among the studies, including hydrocortisone (*Chi*^2^ = 3.38, *I*^2^ = 11%) and dexamethasone (*Chi*^2^ = 3.57, *I*^2^ = 44%). However, heterogeneity exists among the studies in methylprednisolone (*Chi*^2^ = 12.38, *I*^2^ = 52%) (Fig. 5).

**Fig. 6:**
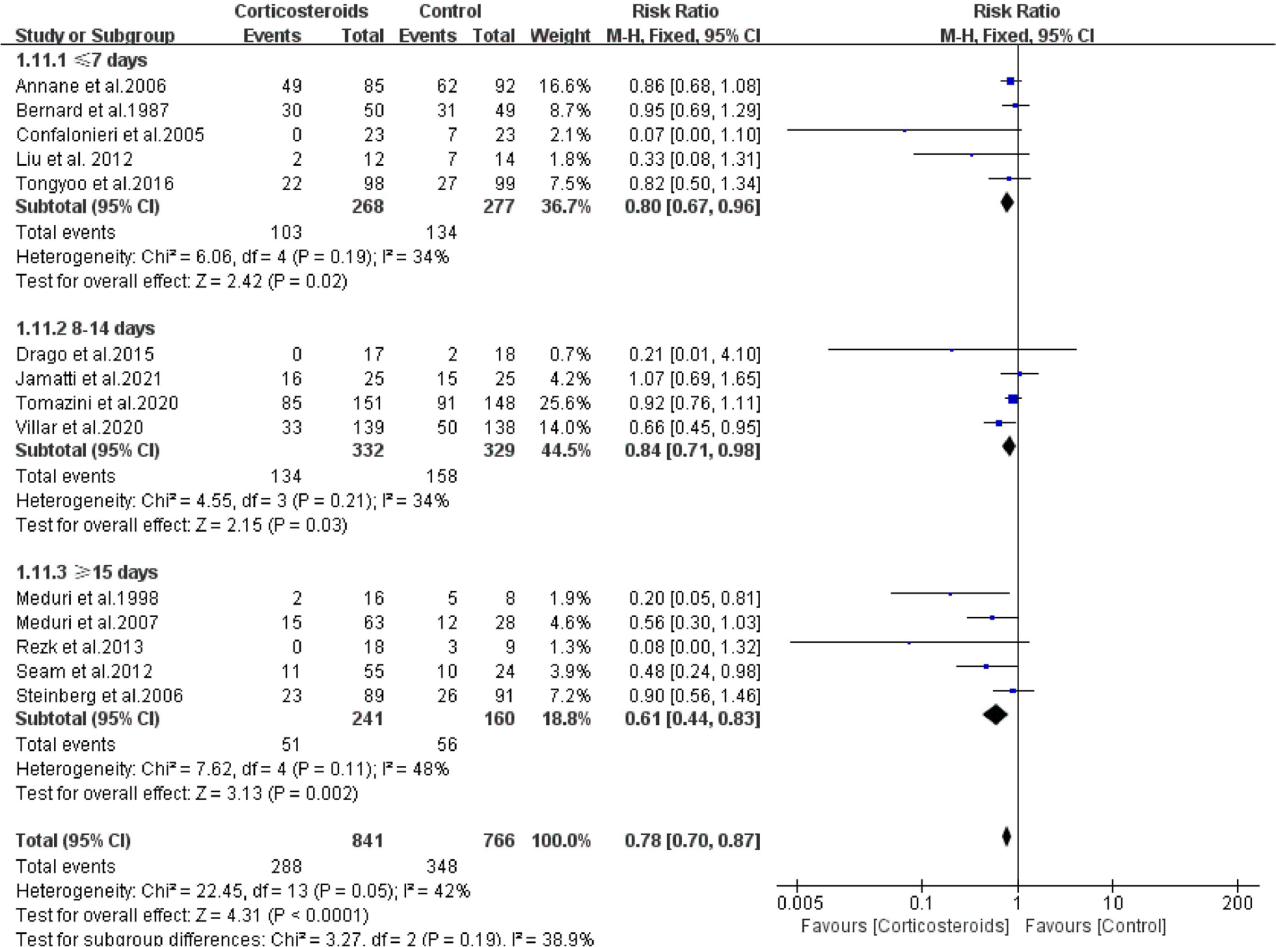
The effect of corticosteroids on Mortality at 28 days. Studies subdivided by treatment duration of corticosteroids

Treatment duration: Our data suggested that glucocorticoids use ≤ 7 days (RR = 0.80; 95% CI: 0.67–0.96; *P* = 0.02), 8–14 days (RR = 0.84; 95% CI: 0.71–0.98; *P* = 0.03) and ≥ 15 days (RR = 0.61; 95% CI: 0.44–0.83; *P* < 0.01) could significantly reduce risk of mortality among patients using corticosteroid. No heterogeneity among studies was found in ≤ 7 days (*Chi*^2^ = 6.06, *I*^2^ = 34%), 8–14 days (*Chi*^2^ = 4.55, *I*^2^ = 34%) and ≥ 15 days (*Chi*^2^ = 7.62, *I*^2^ = 48%) (Fig. 6).

Dose: Since methylprednisolone is known to have a very high efficacy, a secondary analysis of methylprednisolone dose was performed. We found that a high-dose of methylprednisolone (> 2 mg/kg/d) did not affect patient mortality (RR = 0.95; 95% CI: 0.69–1.29; *P* = 0.74), whereas a low-dose of methylprednisolone (≤ 2 mg/kg/d) could significantly reduce patient’s risk of mortality (RR = 0.53; 95% CI: 0.33–0.86; *P* = 0.01). No significant heterogeneity was found among studies, including low-dose (*Chi*^2^ = 8.15, *I*^2^ = 39%) (Additional file 1: Fig. S6).

Treatment duration: Based on the methylprednisolone data, compared to short treatment duration (≤ 14 days) (RR = 0.90, 95% CI: 0.65–1.23; *P* = 0.49), long treatment duration (≥ 15 days) was more effective to reduce the risk of mortality (RR = 0.61, 95% CI: 0.44–0.83; *P* = *P* < 0.01). No significant heterogeneity among studies was found in either the short treatment duration (*Chi*^2^ = 1.04, *I*^2^ = 4%) and long treatment duration (*Chi*^2^ = 7.62, *I*^2^ = 48%) (Additional file 1: Fig. S7).

#### Publication bias

Publication bias was assessed using Egger’s test (*P* = 0.052) and Begg’s test (*P* = 0.119), and no evidence was found (Additional file 1: Fig. S8).

## Discussion

Compared to a previous meta-analysis published in 2021 [21], the present study has several advantages as it has six additional RCTs and contains children and participants with COVID-19. Moreover, the sample size of our study was relatively large and the research was of high quality. In the present study, we found that corticosteroids could significantly reduce the 28-day mortality of ARDS patients, which is inconsistent with several previous meta-analyses [19,26,41]. Corticosteroid use can improve ICU mortality, in-hospital mortality, and ventilation-free days at 28 days, and these beneficial effects have also been confirmed by other studies [21]. In addition, glucocorticoid use could potentially increase the risk of hyperglycemia (but not gastroduodenal bleeding) among ARDS patients, which is consistent with findings by Fang et al. [31]. Therefore, we recommend that blood glucose should be monitored in ARDS patients who receive glucocorticoids. Subgroup analysis showed that the efficacy of corticosteroids in ARDS patients was associated with several variables, such as age, etiologies, corticosteroid type and dosage, and courses of treatment. Therefore, ARDS patients could benefit from steroid therapy.

Various efficacy of glucocorticoid treatment have been observed in children and adults with ARDS. First, there are a limited number of studies investigating the role of glucocorticoid therapy for ARDS in children. Most treatment choices for children with ARDS are made based on the experience of adult ARDS treatment, such as mechanical ventilation, prone position ventilation, drug therapy, and others [42]. Second, the corticosteroid treatment is not recommended in children with ARDS according to the current guidelines[43]. Our findings in this study confirmed that corticosteroid therapy is beneficial to adults with ARDS, but not children. An RCT of children with ARDS (n = 35) was included in our study [23]. The results showed that although glucocorticoids increased the oxygenation index (PaO_2_/FiO_2_) on the 8th and 9th days, they did not reduce mortality. The different efficacy of glucocorticoid treatment in children with ARDS compared to adults with ARDS may be explained by the different disease patterns, immune responses, and lung growth/development abilities [23]. Therefore, current evidence does not support corticosteroid treatment for the management of children with ARDS, and further investigation is required to fill this gap in the research. In addition, previous studies have revealed that corticosteroids may affect growth and metabolism [44]. As a result, caution is required for the indication of corticosteroid therapy.

The effect of corticosteroid treatment is different between non-COVID-19 and COVID-19 patients. In non-COVID-19 ARDS patients, corticosteroids can reduce mortality in the experimental group, while no improvements in mortality were observed in patients with COVID-19. Similar results were obtained by Baek et al. [45,46]. However, this finding conflicts with the current recommendation of steroid treatment for ARDS caused by COVID-19 [47]. The heterogeneous results may be caused by the disease severity and the different types of glucocorticoids used in patients with COVID-19.

Each corticosteroid treatment identified had a varying effect on ARDS patients. In the past, when assessing the efficacy of corticosteroids, most studies target the dose and duration of corticosteroid treatment [20,21], and ignore the specific type of corticosteroid. In the subgroup analysis of our study, it was found that methylprednisolone and hydrocortisone could reduce the mortality of ARDS patients, with methylprednisolone performing particularly well. This effect difference may be explained by the fact that methylprednisolone has a longer in vivo residence time and relatively high concentration in the lung when compared to hydrocortisone [48]. However, dexamethasone does not show a significant effect, although it appears to reduce mortality. In general, our data suggests that different types of glucocorticoids could lead to variable effects on the outcome of ARDS patients. Selecting the appropriate glucocorticoid treatment should be dependent on the patient’s condition.

The use of corticosteroids is always a complex problem in practice. Long-term administration could increase the risk of hyperglycemia and make the disease worse [31]. Hence, an appropriate duration of corticosteroid treatment is required. Our data showed that corticosteroid treatment with a duration of ≤ 7 days, 8–14 days, or ≥ 15 days, could significantly reduce the mortality of ARDS patients. To avoid adverse events, a short duration of corticosteroid treatment is therefore recommended based on our findings. However, a low-dose and long-course of methylprednisolone was found to reduce the mortality of the experimental group significantly, and similar results were confirmed in previous studies [22,48].

The recommended duration is inconsistent which could be explained by the heterogeneity due to different corticosteroids used (hydrocortisone and dexamethasone vs methylprednisolone). The metabolic pathway of the two glucocorticoids (hydrocortisone and dexamethasone) is different to that of methylprednisolone [49], resulting in a different conclusion. Meanwhile, the data confirms the importance of selecting the correct corticosteroid. Unfortunately, the choice of corticosteroid for the management of ARDS patients is still uncertain and further assessments are needed to evaluate the indicators (such as clinical phenotype, immunology, and ARDS treatment) for each corticosteroid.

### Limitations

Although several interesting findings were found, the study also has some limitations. First, due to the nature of systematic reviews and meta-analyses, heterogeneity is an inherent disadvantage, and several variables (such as treatment protocol, dosage, and types of corticosteroids used) varied widely across the studies. Second, the sample size is small, adverse events are infrequent, and the insufficient data makes the assessment of adverse events between subgroups difficult. In addition, due to the short follow-up period of RCTs, the long-term adverse effects of corticosteroid use were neither evaluated nor recorded. Further observational studies are needed to provide more details regarding the potential long-term effects of corticosteroid use in ARDS patients.

## Conclusions

Corticosteroids are an effective approach to reduce the risk of death in ARDS patients. However, this effect is associated with age, non-COVID-19 diseases, and methylprednisolone and hydrocortisone use. Therefore, evidence suggests patients with age ≥ 18 years and non-COVID-19 should be encouraged during the corticosteroid treatment. However, due to substantial differences in the use of corticosteroids among these studies, questions still remain regarding the dosage, optimal corticosteroid agent, and treatment duration in patients with ARDS. Therefore, further investigation is required.

## Supplementary Information

**Additional file 1: Table S1.** Search strategy. **Figure S1.** Assess risk of bias. A. Risk of bias summary; B. Risk of bias graph. **Figure S2.** The effect of corticosteroids on mortality in ICU, in hospital and 60-days. A. Mortality in ICU; B. Mortality in hospital; C. Mortality at 60-days. **Figure S3.** Ventilation-free days at day 28 among patients with ARDS**. Figure S4.** Duration of hospital stay and ICU stay among patients with ARDS. A. ICU stay; B. Hospital stay. **Figure S5.** Adverse events among patients with ARDS. A. Hyperglycemia; B. Gastroduodenal bleeding. **Figure S6.** The effect of corticosteroids on mortality at 28 days. Studies subdivided by different dosage of methylprednisolone. **Figure S7.** The effect of corticosteroids on mortality at 28 days. Studies subdivided by different treatment duration of methylprednisolone. **Figure S8.** Assess the potential publication bias. (A) Egger's test; (B) Begg’s test.

**Additional file 2: Table S2.** Characteristics of the 14 randomized clinical trials of corticosteroids in patients with ARDS.

## Data Availability

The data not published within the present study is available in a public repository for any of the participating studies. Requests for information on procedures, statistical analysis and formal data requests can be submitted to investigators (X.C.; S.L.)

## Abbreviations

ARDS: Acute respiratory distress syndrome
Chi^2^: Chi square
CI: Confidence interval
COVID-19: Coronavirus disease 2019
ICU: Intensive care unit
MD: Mean difference
RCTs: Randomized controlled trials
RR: Risk ratios
