# Supplementary material for: Safety and efficacy of corticosteroids in ARDS patients: a systematic review and meta-analysis of RCT data
Source: Respir Res. 2022 Nov 4;23:301. doi: 10.1186/s12931-022-02186-4 (PMC9635104; doi:10.1186/s12931-022-02186-4)

**Supplementary Table 1.** Search strategy.

| **MEDLINE(R)** | | |
| --- | --- | --- |
| 1 | exp Acute Respiratory Distress Syndrome | 37899 |
| 2 | exp Adrenal Cortex Hormones | 413272 |
| 3 | exp STEROIDS | 893405 |
| 4 | (Adrenal Cortex Hormone* or adrenocortical hormone* or adrenocorticosteroid* or Corticosteroid* or Corticoid* or steroid* or glucocort* or cortisone* or hydrocortisone* or Cortisol or Epicortisol or Cortifair or Cortril or hydroxyhydrocortisone or oxohydrocortisone or tetrahydrocortisol or dexamethason* or baycuten or dexatopic or sofradex or Methylfluorpreordnisolone or Hexadecadrol or Decameth or Decaspray or Dexasone or Dexpak or Maxidex or Millicorten or Oradexon or Decaject or Decaject or Hexadrol or methylprednisolon* or (methyl adj3 prednisolone) or Metipred or Urbason or Medrol or Betamethasone or Flubenisolone or Betadexamethasone or Celestona or  Cellestoderm or Celeston or Celestone or prednison* or prednisolon* or hydroxyprednisolone or desonide or Predate or Predonine or Di-Adreson-F or DiAdresonF or triamcinolon*).mp. | 768919 |
| 5 | 2 or 3 or 4 | 1306479 |
| 6 | randomized controlled trial.pt. | 560539 |
| 7 | controlled clinical trial.pt. | 94724 |
| 8 | randomized.ab. | 552990 |
| 9 | placebo.ab | 226161 |
| 10 | drug therapy.fs. | 226161 |
| 11 | randomly.ab. | 377423 |
| 12 | trial.ab. | 590140 |
| 13 | groups.ab. | 2319851 |
| 14 | 6 or 7or 8 or 9 or 10 or 11 or 12or 13 | 333626 |
| 15 | exp animals/ not humans.sh | 4968930 |
| 16 | 14 not 15 | 2856312 |
| 17 | (Respiratory Distress Syndrome or Distress Syndrome*, Respiratory or shock lung or Acute Respiratory Distress Syndrome or ARDS).mp. | 53818 |
| 18 | 1 or 17 | 55876 |
| 19 | 5 and 16 and 18 | 1246 |
|  | **EMBASE** |  |
| 1 | exp corticosteroid/ | 1014905 |
| 2 | (Adrenal Cortex Hormone* or adrenocortical hormone* or adrenocorticosteroid* or Corticosteroid* or Corticoid* or steroid* or glucocort* or cortisone* or hydrocortisone* or Cortisol or Epicortisol or Cortifair or Cortril or hydroxyhydrocortisone or oxohydrocortisone or tetrahydrocortisol or dexamethason* or baycuten or dexatopic or sofradex or Methylfluorpreordnisolone or Hexadecadrol or Decameth or Decaspray or Dexasone or Dexpak or Maxidex or Millicorten or Oradexon or Decaject or Decaject or Hexadrol or methylprednisolon* or (methyl adj3 prednisolone) or Metipred or Urbason or Medrol or Betamethasone or Flubenisolone or Betadexamethasone or Celestona or  Cellestoderm or Celeston or Celestone or prednison* or prednisolon* or hydroxyprednisolone or desonide or Predate or Predonine or Di-Adreson-F or DiAdresonF or triamcinolon*).mp. | 1343420 |
| 3 | 1 or 2 | 1414277 |
| 4 | exp respiratory distress syndrome | 89481 |
| 5 | (Respiratory Distress Syndrome or Distress Syndrome*, Respiratory or shock lung or Acute Respiratory Distress Syndrome or ARDS).mp. | 85302 |
| 6 | randomized controlled trial/ | 698992 |
| 7 | crossover procedure/ | 69643 |
| 8 | double blind procedure/ | 192990 |
| 9 | single blind procedure/ | 45420 |
| 10 | (random* or factorial* or crossover* or placebo* or assign* or allocat* or volunteer* or (doubl* adj5 blind*) or (singl* adj5 blind*)).mp. | 2911411 |
| 11 | 6 or 7 or 8 or 9 or 10 | 2911411 |
| 12 | exp animal | 28263263 |
| 13 | human | 23207069 |
| 14 | 12 not 13 | 5056194 |
| 15 | 11 not 14 | 2636313 |
| 16 | 4 or 5 | 102021 |
| 17 | 3 and 15 and 16 | 2219 |
|  | **Cochrane Central Register of Controlled Trials** |  |
| 1 | exp Adrenal Cortex Hormones/ | 28815 |
| 2 | exp STEROIDS/ | 55926 |
| 3 | (Adrenal Cortex Hormone* or adrenocortical hormone* or adrenocorticosteroid* or Corticosteroid* or Corticoid* or steroid* or glucocort* or cortisone* or hydrocortisone* or Cortisol or Epicortisol or Cortifair or Cortril or hydroxyhydrocortisone or oxohydrocortisone or tetrahydrocortisol or dexamethason* or baycuten or dexatopic or sofradex or Methylfluorpreordnisolone or Hexadecadrol or Decameth or Decaspray or Dexasone or Dexpak or Maxidex or Millicorten or Oradexon or Decaject or Decaject or Hexadrol or methylprednisolon* or (methyl adj3 prednisolone) or Metipred or Urbason or Medrol or Betamethasone or Flubenisolone or Betadexamethasone or Celestona or  Cellestoderm or Celeston or Celestone or prednison* or prednisolon* or hydroxyprednisolone or desonide or Predate or Predonine or Di-Adreson-F or DiAdresonF or triamcinolon*).mp. | 96669 |
| 4 | 1 or 2 or 3 | 126017 |
| 5 | exp Respiratory Distress Syndrome,Newborn/ | 1684 |
| 6 | (Respiratory Distress Syndrome or Distress Syndrome*, Respiratory or shock lung or Acute Respiratory Distress Syndrome or ARDS).mp. | 6644 |
| 7 | 5 or 6 | 6732 |
| 8 | 4 and 7 | 983 |

**Supplementary Figure 1.** Assess risk of bias. A. Risk of bias summary; B. Risk of bias graph.

A


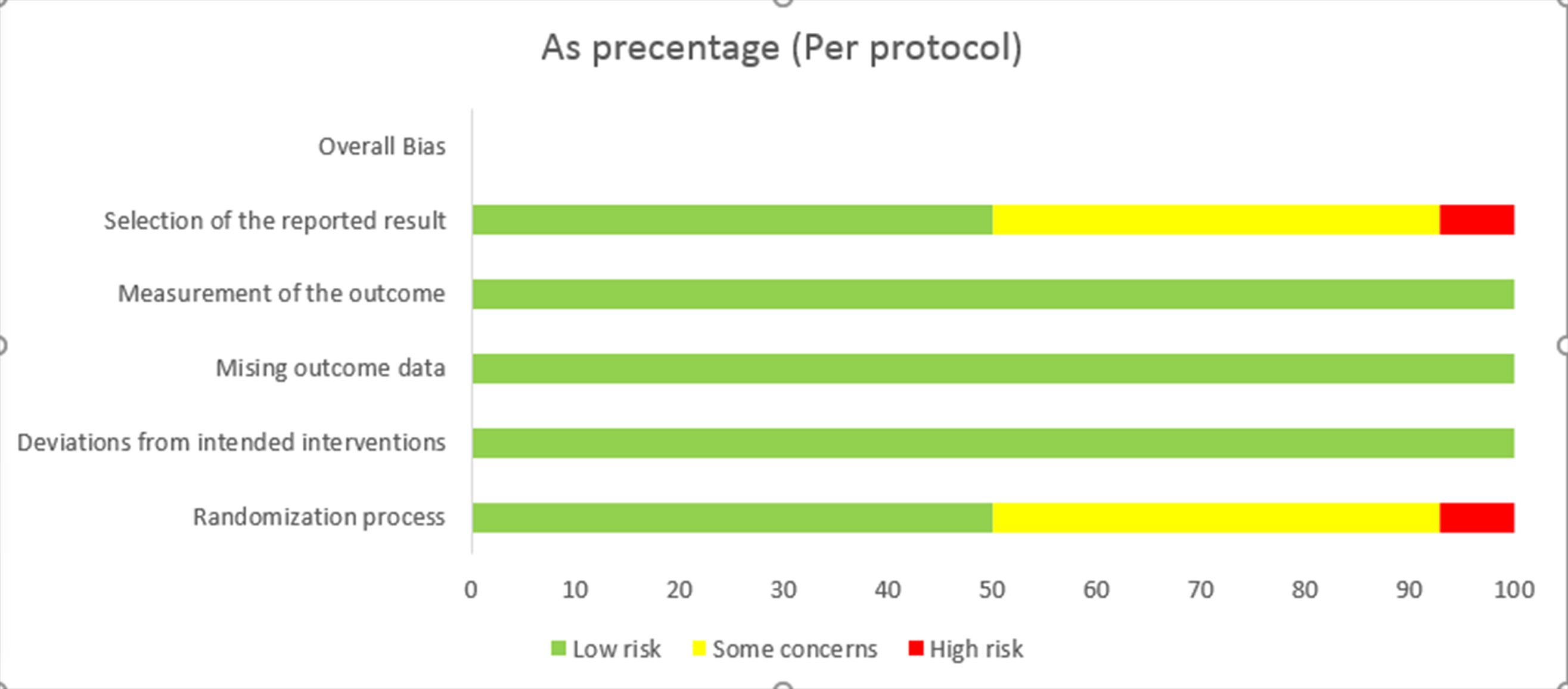


B


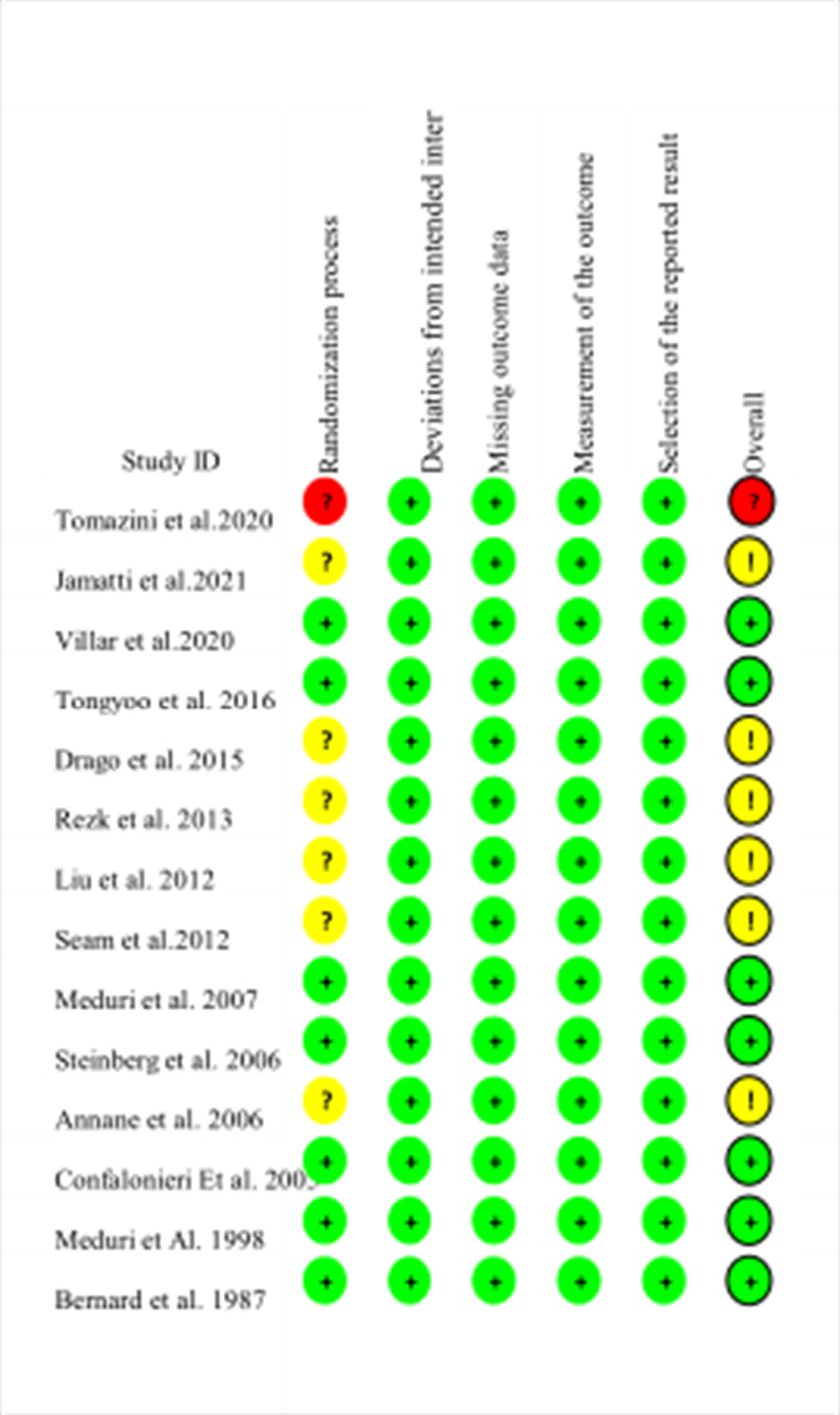


　 Low risk Some concerns High risk

**Supplementary Figure 2.** The effect of corticosteroids on mortality in ICU, in hospital and 60-days. A. Mortality in ICU; B. Mortality in hospital; C. Mortality at 60-days.


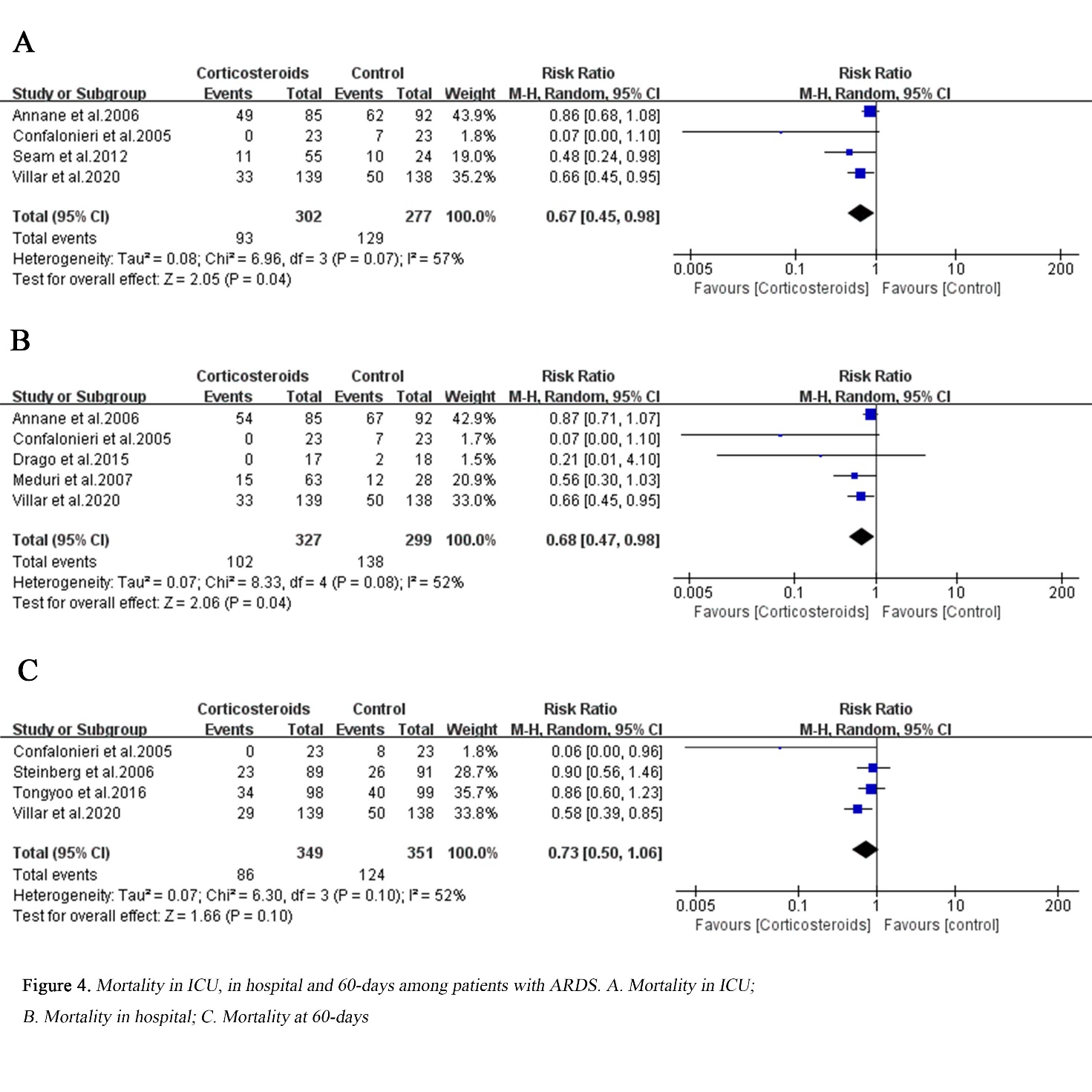


**Supplementary Figure 3.** Ventilation-free days at day 28 among patients with ARDS**.**

**
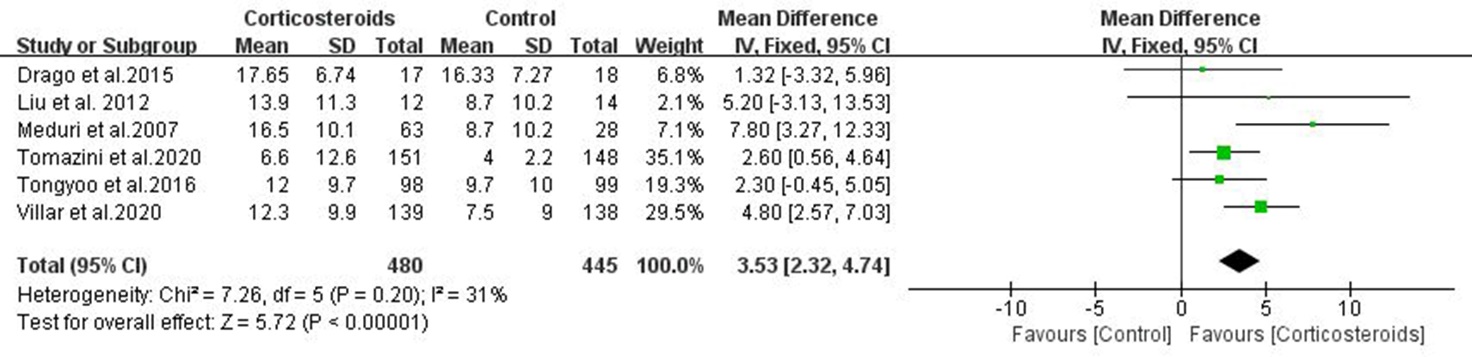
**

**Supplementary Figure 4.** Duration of hospital stay and ICU stay among patients with ARDS. A. ICU stay; B. Hospital stay.


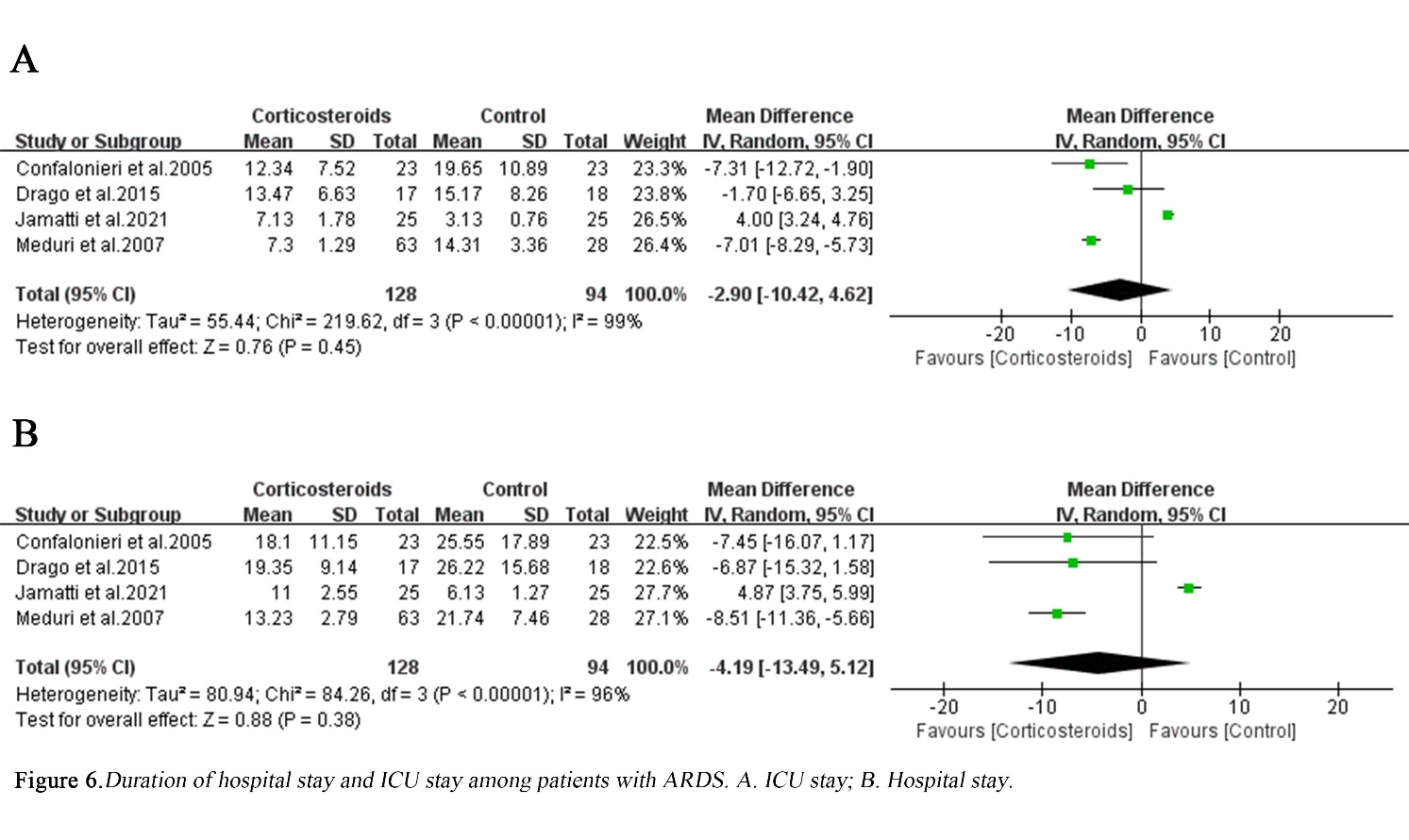


**Supplementary Figure 5.** Adverse events among patients with ARDS. A. Hyperglycemia; B. Gastroduodenal bleeding.


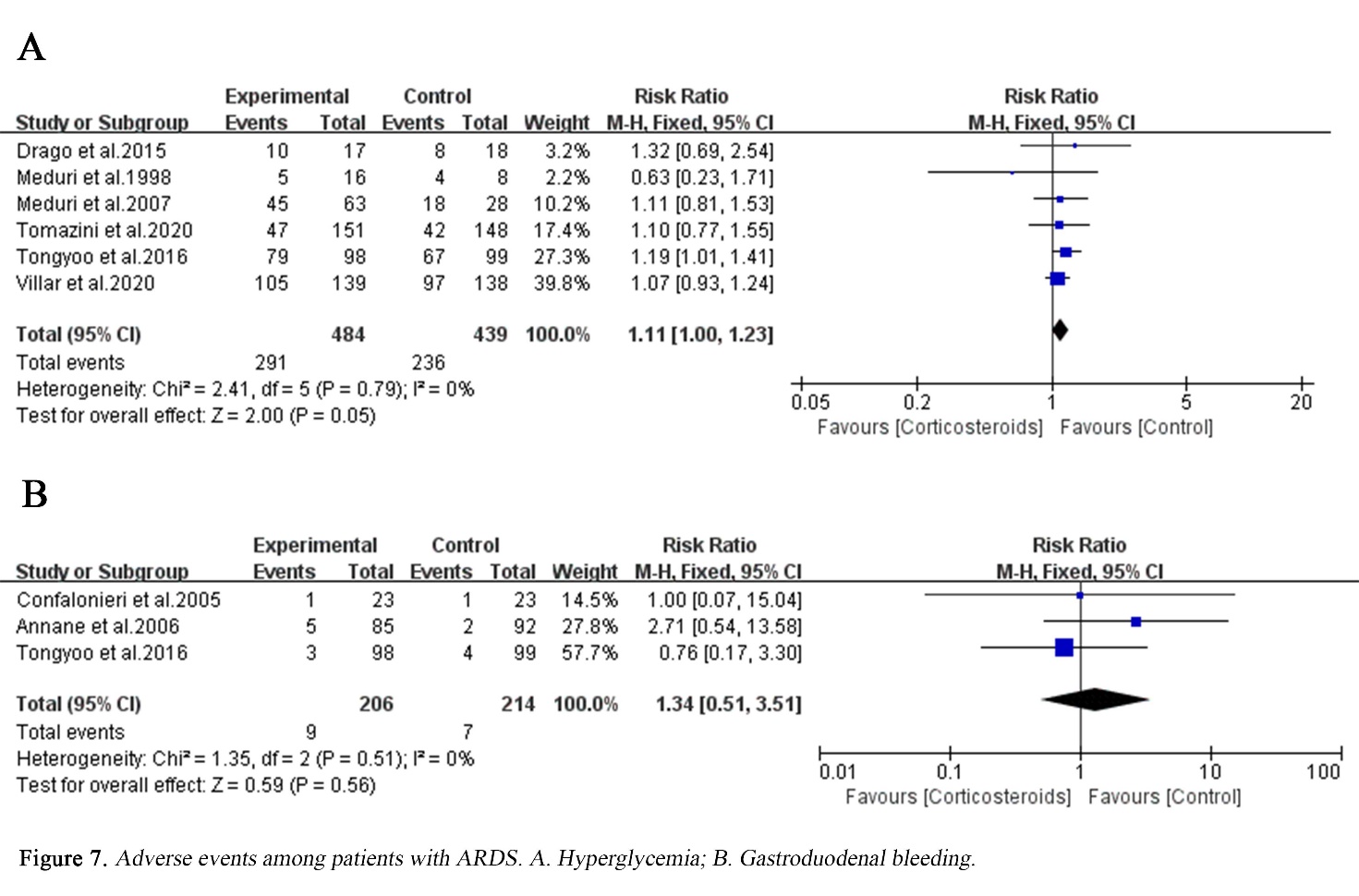


**Supplementary Figure 6.** The effect of corticosteroids on mortality at 28 days. Studies subdivided by different dosage of methylprednisolone.


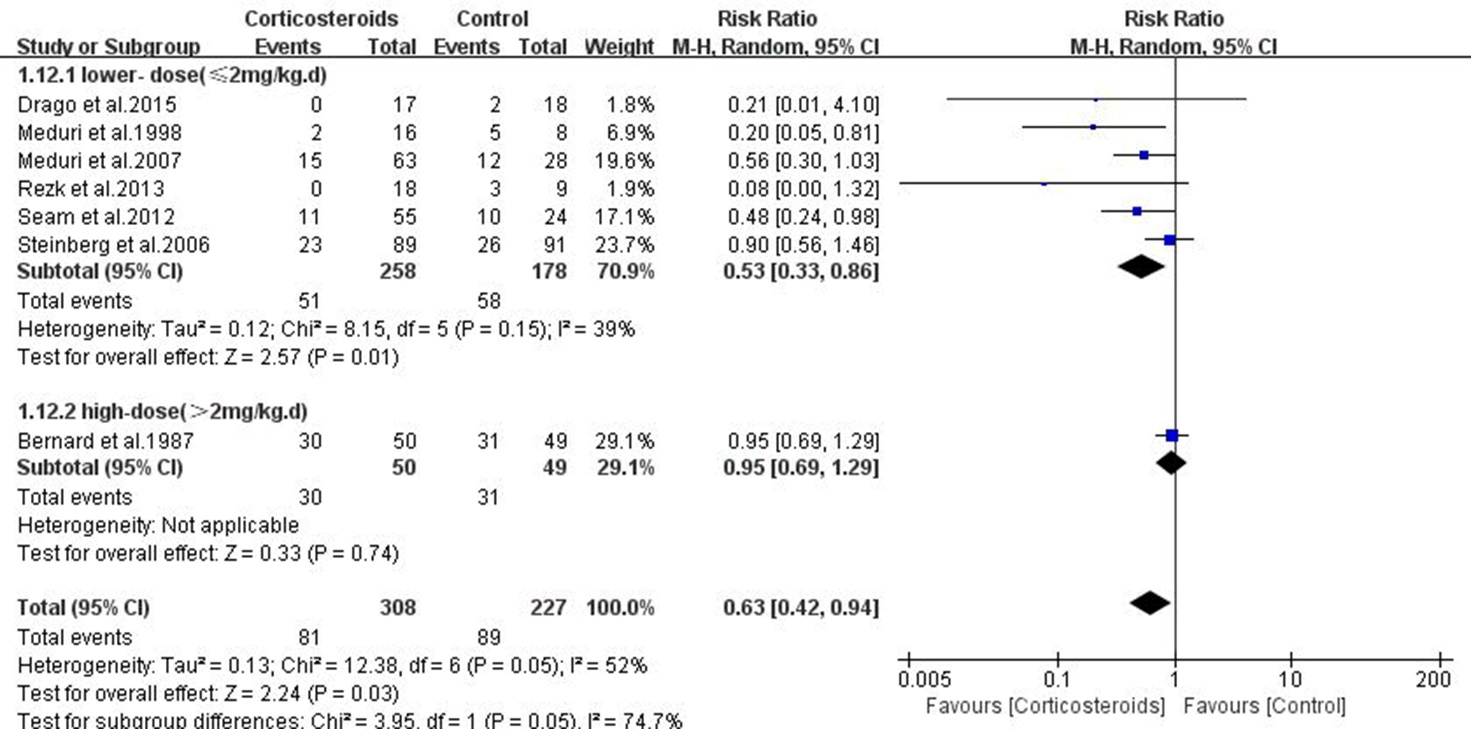


**Supplementary Figure 7.** The effect of corticosteroids on mortality at 28 days. Studies subdivided by different treatment duration of methylprednisolone.


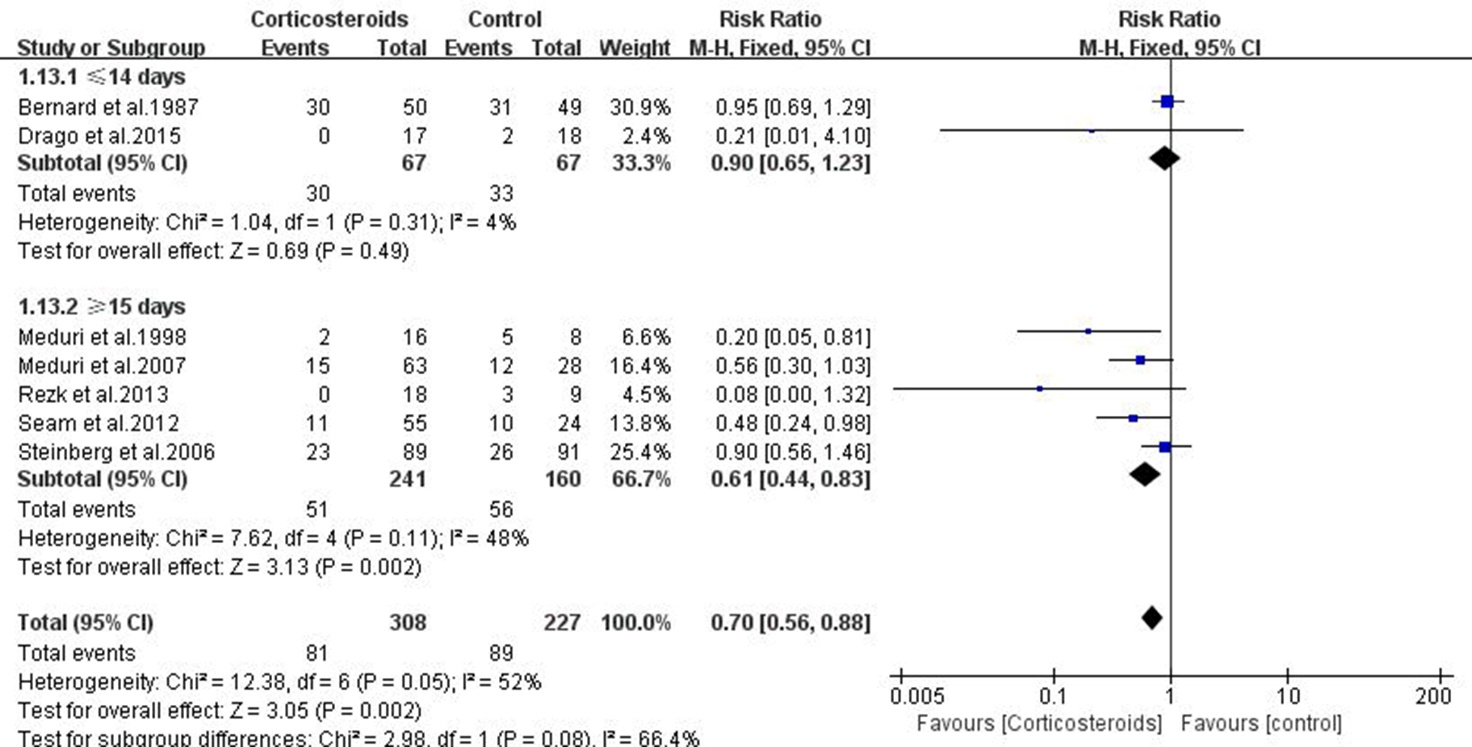


**Supplementary Figure 8.** Assess the potential publication bias. (A) Egger's test; (B) Begg’s test.


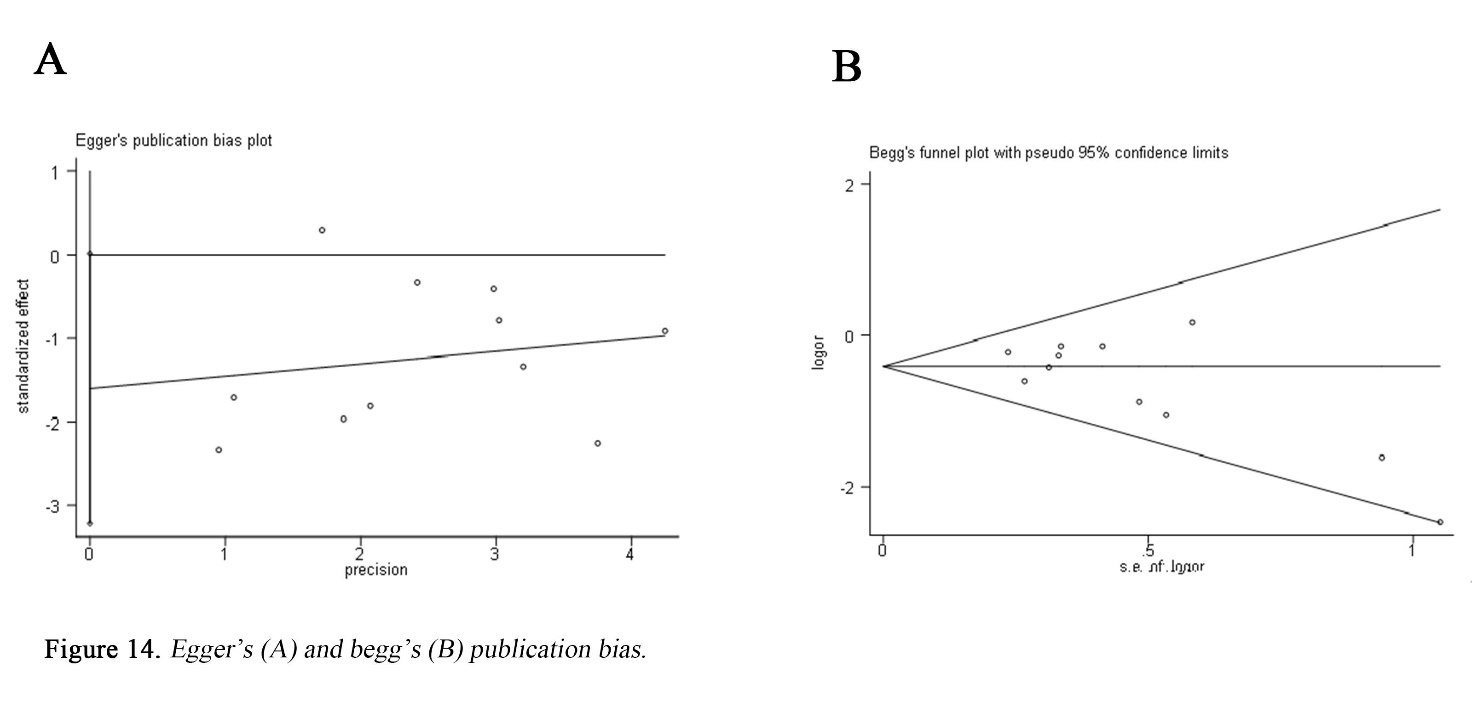

Supplement: Supplementary file 1 — Additional file 1: Table S1. Search strategy. Figure S1. Assess risk of bias. A. Risk of bias summary; B. Risk of bias graph. Figure S2. The effect of corticosteroids on mortality in ICU, in hospital and 60-days. A. Mortality in ICU; B. Mortality in hospital; C. Mortality at 60-days. Figure S3. Ventilation-free days at day 28 among patients with ARDS. Figure S4. Duration of hospital stay and ICU stay among patients with ARDS. A. ICU stay; B. Hospital stay. Figure S5. Adverse events among patients with ARDS. A. Hyperglycemia; B. Gastroduodenal bleeding. Figure S6. The effect of corticosteroids on mortality at 28 days. Studies subdivided by different dosage of methylprednisolone. Figure S7. The effect of corticosteroids on mortality at 28 days. Studies subdivided by different treatment duration of methylprednisolone. Figure S8. Assess the potential publication bias. (A) Egger's test; (B) Begg’s test. [file 12931_2022_2186_MOESM1_ESM.docx]
