# Supplementary material for: Safety and efficacy of corticosteroids in ARDS patients: a systematic review and meta-analysis of RCT data
Source: Respir Res. 2022 Nov 4;23:301. doi: 10.1186/s12931-022-02186-4 (PMC9635104; doi:10.1186/s12931-022-02186-4)
Supplement: Supplementary file 2 — Additional file 2: Table S2. Characteristics of the 14 randomized clinical trials of corticosteroids in patients with ARDS. [file 12931_2022_2186_MOESM2_ESM.docx]

**Supplementary Table 2. Characteristics of the 14 randomized clinical trials of corticosteroids in patients with ARDS.**

| Study No. of Mean Age,  Patients y | Inclusion criteria | Intervention Treatment duration Primary Outcome |
| --- | --- | --- |
| Jamatti et al. 50 CS:61.9  2021 PC:61.8 | (1) Age > 18 years; (2) SARS-CoV-2 infection confirmed by a reverse transcription-polymerase chain reaction test; (3) ratio of partial pressure of oxygen in arterial blood to fraction of inspired oxygen (PaO2/FiO2) between 100 and 300 mmHg; (4) bilateral lung infiltration. | Dexamethasone 20 mg/day 10 days Invasive mechanical  from day 1–5 and then at ventilation and death  10 mg/day from day 6–10. rate |
| Tomazini et al. 299 CS:60.1  2020 PC:2.7 | (1) Adult patients with confirmed or  suspected COVID-19 infection were receiving mechanical ventilation; (2) patients diagnosed with moderate to severe ARDS by the Berlin criteria. | Dexamethasone 20 mg daily 10 days or until 28-day all-cause  from day 1 to day 5, followed by ICU discharge mortality  10 mg daily for 5 days or  until ICU discharge. |
| Villar et al. 277 CS:56  2020 PC:58 | (1) Adult patients were intubated and  Mechanically ventilated;(2) patients diagnosed with ARDS by the AECC definition or the Berlin criteria as moderate to severe ARDS. | Dexamethasone 20 mg once 10 days Ventilator-free days  daily from day1 to day 5, during the first 28  which was reduced to 10 mg days  once daily from day 6 to day  10. |
| Tongyoo et al. 197 CS:64.5  2016 PC:64.3 | Patients diagnosed with ARDS by the AECC definition and the Berlin criteria. | Hydrocortisone 50 mg every 7 days 28-day all-cause  6 h. mortality |
| Drago et al. 35 CS:8.2  2015 PC:3.7 | Acute lung injury/ARDS (based on the American European Consensus  Conference consensus criteria) in children aged 1 month to 18 years who were mechanically ventilated for less than 72 hours. | Methylprednisolone 2 mg/kg 14 days Ventilator-free days  over 15 minutes, Followed  by continuous infusions  1 mg/kg/d on days 1–7, 0.5  mg/kg/d on days 8–10, 0.25  mg/kg/d on days 11 and 12, and  0.125 mg/kg/d on days 13 and 14. |
| Rezk et al. 27 CS:42.7  2013 PC:50.4 | (1) All patients must get criteria of ARDS; (2) All patients must be on mechanical ventilation. (3) Methyl prednisolone must be started randomly in first 48 h. | Methylprednisolone; loading dose 28 days Mortality at day 14  of 1 mg/kg followed by infusion of  1 mg/kg/day on days 1-14, 0.5  mg/kg/day on days 15- 21, 0.25  mg/kg/day from days 22 -25,  0.125 mg/kg/day from days 26 -28. |
| Meduri et al. 91 CS:50.1  2007 PC:53.2 | Adult intubated patients diagnosed with ARDS by the AECC definition. | Methylprednisolone 1 mg/kg bolus 28 days 1-point reduction in  followed by 1 mg/kg (days 1 –14), LIS or successful  0.5 mg/kg (days15 –21), 0.125 extubation day7  mg/kg (days 22 –25), 0.25 mg/day  (days 26 –28). |
| Steinberg et al. 180 CS:49  2006 PC:49.2 | Adult patients diagnosed with ARDS by the AECC definition; (2) patients were intubated and mechanically ventilated for 7 –28 days after the onset of ARDS. | Methylprednisolone 2 mg/kg bolus 25 days 60-day all-cause  followed by 2 mg/kg (days 1 –14)， mortality  1 mg/kg (days 15 –21), tapering  over (days 22 –25). |
| Annane et al. 177 CS:61  2006 PC:59 | (1) Septic shock patients with bilateral infiltrates on chest radiography; (2) PaO2/FIO2 ≤200; (3) PAWP ≤18 mmHg or no clinical evidence of left atrial hypertension. | Hydrocortisone 30 mg IV every 7 days 28-day survival  6 h and 9-fludrocortisone 50ug  once a day. |
| Bernard et al. 99 CS:55  1987 PC:59 | (1) Patients with PaO2 ≤70 mmHg  (FiO2 ≥40%) or PaO2/PAO2 ≤0.3; (2) diffuse infiltrates on chest radiography;(3) PAWP ≤18 mmHg. | Methylprednisolone 30 mg/kg IV 1 day 45-day all-cause  every 6 h. mortality |
| Liu et al. 26 CS:69.8  2012 PC:55.9 | Adults 18 to 80 years of age; fulfils criteria of ARDS according to the AECC (Bernard et. al). | Hydrocortisone 100 mg IV 3 7days Overall mortality  times a day. at day 28 |
| Confalonieri 46 CS:60.4  Et al. 2005 PC:66.6 | Clinical and radiographic evidence of pneumonia with bilateral or multi-lobar involvement and PaO2/FiO2＜250. | Hydrocortisone 200 mg bolus 7 days Mortality  followed by an infusion of 10  mg/h. |
| Meduri et 24 CS:47  Al. 1998 PC:51 | Patients diagnosed with ARDS by the AECC definition. | Methylprednisolone 2 mg/kg 32 days Improvement in (days 1 –14), 1 mg/kg , lung  (days15 –21) 0.5 mg/kg  (days 22-28), 0.25mg/kg  (days 29 – 30), and 0.125 mg/kg  (days 31 -32). |
| Seam et al. 79 CS:49.7  2012 PC:53.9 | Patients in both arms of the clinical study received mechanical ventilation for ARDS based on the standard practice at the time of enrollment. | Methylprednisolone infusion 28 days Biomarkers  (1 mg/kg/day for 14 days and  then tapered over 2 weeks). |
